# Molecular analysis of carbapenem-resistant *Acinetobacter baumannii* isolated from bronchoalveolar lavage fluid in a tertiary hospital in Ningxia, China

**DOI:** 10.3389/fcimb.2026.1752819

**Published:** 2026-03-10

**Authors:** Jing Zhang, Dong Liu, Shan Li, Pengtao Wang, Wei Jia

**Affiliations:** 1 First Clinical Medical College, Ningxia Medical University, Yinchuan, Ningxia, China; 2 Department of Clinical Laboratory Diagnostics, People’s Hospital of Ningxia Hui Autonomous Region, Yinchuan, Ningxia, China; 3 Continuing Education College, Ningxia Medical University, Yinchuan, Ningxia, China; 4 Ningxia Key Laboratory of Clinical and Pathogenic Microbiology, Institute of Medical Sciences, General Hospital of Ningxia Medical University, Yinchuan, Ningxia, China; 5 Center of Medical Laboratory, General Hospital of Ningxia Medical University, Yinchuan, Ningxia, China

**Keywords:** carbapenem-resistant *Acinetobacter baumannii*, multilocus sequence typing, resistance, ST195, virulence

## Abstract

**Purpose:**

Infections caused by clinical carbapenem-resistant *Acinetobacter baumannii* (CRAB) are associated with an increased risk of mortality and present a significant challenge for hospitals worldwide. This study aims to analyze the molecular epidemiological characteristics, drug resistance traits, and virulence features of CRAB isolated from bronchoalveolar lavage fluid (BALF) at a hospital in Ningxia, China.

**Methods:**

We collected clinical characteristic data of patients with isolated strains and conducted statistical analysis. Antibiotic susceptibility testing was conducted using the VITEK-2 compact system. Carbapenemase and virulence genes were examined through PCR and Sanger sequencing. Multilocus Sequence Typing (MLST) was performed according to the Oxford MLST scheme by comparing the obtained sequences with known allele sequences available on the MLST website (http://pubmlst.org/abaumannii/

). The virulence of CRAB was assessed using the *Galleria mellonella* infection assay.

**Results:**

The results indicated that all tested CRAB strains carried the *bla*_OXA-23_ and *bla*_OXA-51_ genes, exhibiting multidrug resistance characteristics while remaining sensitive to polymyxins. MLST typing revealed that ST195 and ST369 strains were the most prevalent, with several other types identified, including ST208, ST136, ST469, ST368, and a rare ST1779. Notably, 94.2% of CRAB belonged to Global clone 2. Significant clinical differences were observed between ST195 and non-ST195 infection cases. Virulence assessment results indicated that 71 strains (58.6%) exhibited high virulence characteristics. Additionally, virulence factors such as *ompA, adeH, pgaA, abal, BasJ*, and *plcD* were detected in all tested strains, confirming an evolutionary trend towards high virulence in CRAB, which poses a serious threat to clinical treatment and patient prognosis.

**Conclusion:**

The emergence of highly virulent multidrug-resistant CRAB strains in the Ningxia region has increased a clinical burden, highlighting the importance of clinical surveillance and diagnosis of these strains.

## Introduction

*Acinetobacter baumannii* (*A. baumannii*), an aerobic, Gram-negative opportunistic pathogen commonly found in hospital settings, can lead to various infections, including ventilator-associated pneumonia, skin and wound infections, bacteremia, meningitis, and urinary tract infections (Lee et al., 2017; Mea et al., 2021). The organism’s resistance to various antibiotics, particularly carbapenems, which are a crucial class of *β*-lactam antibiotics frequently regarded as the last line of treatment for infections caused by *A. baumannii*, complicates treatment options (Ramirez et al., 2020; Müller et al., 2023). This rise can be attributed to the widespread use of broad-spectrum antibiotics, with carbapenem resistance rates exceeding 30% to 90% in regions such as Asia and Latin America (Wong et al., 2017; Shields et al., 2023). According to data from the China Antimicrobial Surveillance Network, the resistance rates of *A. baumannii* to meropenem and imipenem have risen from 39% and 31% in 2005 to 73.7% and 73.4% in 2023, respectively (http://www.chinets.com/). Previous studies have demonstrated that the prevalence of carbapenem-resistant *Acinetobacter baumannii* (CRAB) in sputum cultures from ICUs is significantly higher than that in non-ICUs (Neves et al., 2016; He et al., 2020). In China, the detection rate of CRABin intensive care units (ICUs) has been reported to be 71.4% (Liu et al., 2022). A study from Jiangxi province, China, indicates that the detection rate of CRAB isolated from blood culture in the region has increased from 33.3% in 2020 to 76.5% in 2024 (Liu et al., 2025b). Given China’s high population density and mobility, coupled with a significantly higher percentage of hospitalized patients receiving antibiotic prescriptions compared to the global average, there has been a dramatic increase in carbapenem resistance in China over the past few decades (Zhang et al., 2017). While CRAB has been documented in China, there is a notable lack of data regarding its prevalence in the Ningxia region of Northwest China. Furthermore, the acquisition of virulence genes by CRAB has led to the emergence of hypervirulent CRAB (hv-CRAB), a clone that has been rarely reported. Therefore, it is imperative to conduct a comprehensive study to analyze the prevalence of CRAB and hv-CRAB in this area.

MLST of *A. baumannii* was identified by sequencing the conserved regions of seven housekeeping genes located in the 16S rRNA ribosome: *gltA*, *gyrB*, *gdhB*, *recA*, *cpn60*, *gpi*, and *rpoD* (Bartual et al., 2005). The CRAB strains are classified into two major globally clones, referred to as Global Clone 1 (GC1) and Global Clone 2 (GC2). Currently, sequence types ST191, ST195, and ST208 represent the most prevalent CRAB strains in China, all of which are classified within the GC2 complex (Chang et al., 2015; Jiang et al., 2019; Tian et al., 2024). Furthermore, additional STs, such as ST369, ST1336, ST136, ST138, ST75, and ST381, have also been documented in China (Ruan et al., 2013; Huang et al., 2023; Zhang et al., 2025). This suggests that more new sequence types will likely be reported in the future, indicating the genetic evolution of CRAB, which may result in the emergence of new CRAB types.

The emergence of hv-CRAB is attributed to the co-presence of resistance and virulence genes. The primary mechanism of carbapenem resistance in *A. baumannii* is the hydrolysis of carbapenems by class D *β*-lactamases (CHDLs), including *bla*_OXA-23_, *bla*_OXA-24_, *bla*_OXA-51_, and *bla*_OXA-58_ (Brown and Amyes, 2005; Poirel and Nordmann, 2006; Lin and Lan, 2014; Nowak and Paluchowska, 2016). Additionally, class B metallo-*β*-lactamases, such as *bla*_NDM_, *bla*_VIM_, *bla*_IMP_, and *bla*_SIM_ (Poirel and Nordmann, 2006; Gomaa et al., 2017), also contribute to carbapenem resistance, while class A KPC and GES *β*-lactamases are rare reported (Jeon et al., 2015; Müller et al., 2023). A study conducted in Zhejiang, China, revealed that 99.3% of CRAB isolates obtained from ICUs harbored *bla*_OXA-23_ (Doughty et al., 2023), which is the most commonly class D carbapenemase gene in China (Li et al., 2023). Furthermore, the pathogenicity of *A. baumannii* is significantly influenced by the virulence genes present in its genome, which include outer membrane proteins (such as OmpA), capsular polysaccharides, iron acquisition systems, outer membrane vesicles, secretion systems, phospholipases, and regulatory systems such as H-NS and two-component systems (Lee et al., 2017; Karampatakis et al., 2024; Lucidi et al., 2024). Overall, the emergence of hv-CRAB complicates clinical treatment and poses a more serious threat to patients.

Respiratory infections are among the most prevalent infectious diseases affecting the respiratory system (Tang et al., 2018a). The irrational use of antimicrobial drugs has led to a significant issue of antibiotic resistance in China, contributing to treatment failures in lung infections caused by pathogens (Tang et al., 2018b). The susceptibility of sputum samples to contamination by oral colonizing bacteria complicates the determination of whether the strains isolated from sputum cultures indicate infection, colonization, or contamination. In contrast, bronchoalveolar lavage fluid (BALF), which is a sterile fluid specimen obtained directly from the site of pulmonary infection, minimizes contamination interference and is regarded as a reliable method for identifying the bacterial etiology of lung infections (Escribano Montaner et al., 2018). Consequently, our study investigates the distribution of CRAB isolated from clinical bronchoalveolar lavage fluid in a tertiary hospital in Ningxia, while also identifying the clinical characteristics, resistance profiles, and virulence traits of CRAB. This research offers clinical insights for the monitoring, prevention, and treatment of CRAB in the region.

## Materials and methods

### Strains collection and identification

A total of 121 CRAB isolates were obtained from BALF samples at a tertiary hospital in Ningxia from 2018 to 2023. All strains were identified using MALDI-TOF mass spectrometry. According to the Clinical and Laboratory Standards Institute (CLSI) guidelines (Clinical and Laboratory Standards Institute, 2020), CRAB is defined by its resistance to either imipenem or meropenem. Basic clinical information of the patients was systematically gathered and analyzed retrospectively. This study received ethical approval from the Medical Science Ethics Institutional Review Board of the General Hospital of Ningxia Medical University (KYLL-2025-0183, approved on January 31, 2025).

### Antimicrobial susceptibility testing

All strains underwent antimicrobial susceptibility testing, utilizing *Escherichia coli* ATCC 25922 and *Pseudomonas aeruginosa* ATCC 27853 as reference strains for internal quality control. The susceptibility tests were performed using the BioMérieux VITEK 2 automated susceptibility testing system. Results were interpreted according to the breakpoint criteria established by the Clinical and Laboratory Standards Institute (CLSI) in 2020. The resistance profiles of the clinical isolates were analyzed against 15 common antibiotics, including imipenem, meropenem, minocycline, piperacillin, ticarcillin-clavulanic acid, tobramycin, piperacillin-tazobactam, levofloxacin, trimethoprim-sulfamethoxazole, tigecycline, ciprofloxacin, ceftazidime, colistin, cefoperazone-sulbactam, and cefepime.

### Detection of virulence and antibiotic-resistance genes

To detect resistance and virulence genes in CRAB isolates, polymerase chain reaction (PCR) assays were conducted utilizing conventional PCR amplification techniques. Total genomic bacterial DNA was extracted using a bacterial DNA extraction kit (DP302, Tiangen Biotechnology, Beijing, China). The primers of carbapenemase genes (*bla*_OXA-23_, *bla*_OXA-51_, *bla*_VIM_, *bla*_OXA-24_, *bla*_OXA-58_, *bla*_GES_, *bla*_NDM_, *bla*_IMP_, and *bla*_KPC_), as well as eight virulence genes (*ompA, csuA, pgaA, adeH, abaI, basJ, plcD*, and *ptk*) were listed in **Table 1**. The PCR amplification were performed using conditions described by Neil Woodford et al (Woodford et al., 2006). All PCR products were subjected to Sanger sequencing to identify the various gene subtypes.

**Table 1:** Primer sequencesfor virulence and antibiotic-resistance genes.

Genes	Forward (5'-3')	Reverse (5'-3')	Length of PCR products
*bla*_OXA23_	GATCGGATTGGAGAACCAGA	ATTTCTGACCGCATTTCCAT	501
*bla*_OXA-51_	TAATGCTTTGATCGGCCTTG	TGGATTGCACTTCATCTTGG	353
*bla*_VIM_	GTTTGGTCGCATATCGCAAC	AATGCGCAGCACCAGGATAG	390
*bla*_OXA24_	GGTTAGTTGGCCCCCTTAAA	AGTTGAGCGAAAAGGGGATT	246
*bla*_OXA-58_	AAGTATTGGGGCTTGTGCTG	CCCCTCTGCGCTCTACATAC	599
*bla*_GES_	GCGCTTCATTCACGCACTAT	GCGTAATCTCTCTCCTGGGC	753
*bla*_NDM_	GGTTTGGCGATCTGGTTTTC	CGGAATGGCTCATCACGATC	621
*bla*_IMP_	CTACCGCAGCAGAGTCTTTG	AACCAGTTTTGCCTTACCAT	587
*bla*_KPC_	TGTAAGTTACCGCGCTGAGG	CCAGACGACGGCATAGTCAT	367
*ompA*	CGCTTCTGCTGGTGCTGAAT	CGTGCAGTAGCGTTAGGGTA	531
*csuA*	GGAACTATAGATTTTGGTGAAGC	ACCCTTAGATATACGACTACC	348
*pgaA*	GCTAAAGATCAGTTGTCGAAG	TTCAGCAAAGCTTTCGGCATC	360
*adeH*	CAACTGAATGAACTTGAACAG	GCTGCGTTGACACTACTTGC	291
*abaI*	GTGGCTCAAGACAGAGAATC	ACGTTCTACTCCAAGAGGAG	297
*basJ*	TCATCAGAATTCCAAGGTGTGC	TTCTAACCATTCAGCTTCAGC	300
*plcD*	GCGCTTATTGGTGGGCGCAAT	CTGAACGGTGGCTTGTTGATAATG	246
*ptK*	ATGAACCAAAATACTAATACCG	GTGTATTCAGTTTTATATTCAG	386

### Multilocus sequence typing identification

Strains were typed according to Oxford scheme of *A.baumannii*, using PCR conditions and primers that have been described previously (Bartual et al., 2005). Oxford MLST scheme by comparing the sequences with known allele sequences available on the MLST website (http://pubmlst.org/abaumannii/). This scheme utilizes seven housekeeping genes (*cpn60, gpi, gltA, gyrB, recA, gdhB*, and *rpoD*) for PCR amplification followed by Sanger sequencing. Following the acquisition of the allele and its subsequent classification as MLST type, the data were analyzed using GrapeTree software (https://doi.org/10.1101/gr.232397.117) to construct a phylogenetic tree based on the seven housekeeping genes using the maximum-likelihood method.

### *Galleria mellonella* infection assay

*In vivo* infection assays using *Galleria mellonella* (*G. mellonella*) were conducted following the methodology described by Josephine et al (Hubloher et al., 2023). *G. mellonella* larvae, approximately 20 mm in length and weighing about 250 mg, were procured from Tianjin Huiyude Biotech Company (Tianjin, China) for use as experimental samples. *A. baumannii* was washed to eliminate the culture medium and subsequently resuspended in sterile physiological saline. For each infection experiment, a total of 10 µl of the cell suspension (1 × 10^6 CFU) was injected into the last proleg of the selected larvae, with 10 larvae injected for each strain. The negative control group received an injection of 10 µl of sterile physiological saline, and ATCC19606 (Yao et al., 2024) served as a non-hypervirulent control strain. Following incubation in the dark at 37 °C for 7 days, the survival percentage of the larvae was assessed. All experiments mentioned above were repeated three times independently.

### Statistical analysis

For continuous variables, we first conducted a normality test. If each group satisfied the normality assumption, we used means and standard deviations for statistical description, with t-tests for comparisons between two groups and one-way ANOVA for comparisons among multiple groups. If the normality assumption was not satisfied, we used medians (and interquartile ranges) for statistical description and employed non-parametric tests (Mann-Whitney U test) for comparisons between two groups. For categorical variables, we utilized chi-square tests or Fisher’s exact tests. All significance tests were two-tailed, with a *p*-value of < 0.05 indicating significance. This study utilized SPSS version 27 (SPSS, Chicago, IL) for calculations.

## Results

### Clinical characteristics of 121 patients with CRAB

This study collected samples of CRAB from bronchoalveolar lavage fluid obtained from patients at Ningxia Medical University General Hospital between 2018 and 2023. A statistical analysis of the basic clinical information of patients revealed that males constituted the majority, with 94 cases (77.7%), while females accounted for 27 cases (22.3%). The average age was 56.60 ± 19.43 years, and the average length of hospitalization was 24 days (15-36.5). After treatment, 53 patients (43.8%) recovered well. Laboratory examination of related factors showed that the C-reactive protein (CRP) level was 38 (13-68) mg/L, white blood cell count (WBC) was 12.24 ± 6.25 × 10^9/L, erythrocyte sedimentation rate (ESR) was 25 (12-46) mm/h, hemoglobin was 112.57 ± 27.39 g/L, platelet count was 212 (129.5-295) × 10^9/L, procalcitonin (PCT) was 0.51 (0.22-2.33) ng/mL, and interleukin-6 (IL-6) was 120 (38.48-210.76) pg/mL. All these elevated inflammatory factors indicates severe bacterial infection. The patients included in this study were sourced from various departments: the Intensive Care Unit (ICU) accounted for 87 patients (71.9%), the Department of Respiratory and Critical Care Medicine had 15 patients (12.3%), and the Neurological Care Unit (NCU) consisted of 12 patients (9.9%). Additionally, patients from several other departments were also included. Among these patients, 28 (23.1%) were diagnosed with hypertension, 15 (12.3%) with diabetes, 16 (13.2%) with other diseases, while 62 (51.2%) had no underlying conditions. These findings are summarized in **Table 2**.

**Table 2:** Basic clinical characteristics of the patients.

Clinical features	*n* = 121
Age, year, mean (±SD)	56.60 ± 19.43
Gender, male, %	94 (77.7%)
Hospitalization stay, days, median (IQR)	24 (15-36.5)
Outcome, recovered, N (%)	53 (43.8%)
Laboratory features	
CRP, mg/L, median (IQR)	38 (13-68)
WBC, 10^9/L, mean (±SD)	12.24 ± 6.25
NEUT, %, mean (±SD)	83.59 ± 7.36
LYM, %, median (IQR)	8.32 (5.33-13.38)
ESR, mm/h, median (IQR)	25 (12-46)
Hemoglobin, g/L, mean ± SD	112.57 ± 27.39
Platelet count, 10^9/L, median (IQR)	212 (129.5-295)
PCT, ng/mL, median (IQR)	0.51 (0.22-2.33)
IL-6, pg/mL, median (IQR)	120 (38.48-210.76)
Clinical Department,N(%)	
Intensive Care Unit	87 (71.9%)
Emergency Department	4 (3.3%)
Department of Respiratory and Critical Care Medicine	15 (12.3%)
Neurological Care Unit	12 (9.9%)
Gastrointestinal Surgery	2 (1.6%)
Oral and maxillofacial surgery	1 (0.8%)
Combined underlying diseases,N(%)	
Hypertension	28 (23.1%)
Diabetes mellitus	15 (12.3%)
Cancer	2 (1.6%)
Cardiac disease	4 (3.3%)
COPD	6 (4.9%)
COVID	4 (3.3%)
No underlying diseases	62 (51.2%)

### MLST of 121 CRAB isolates

**Figure 1:**
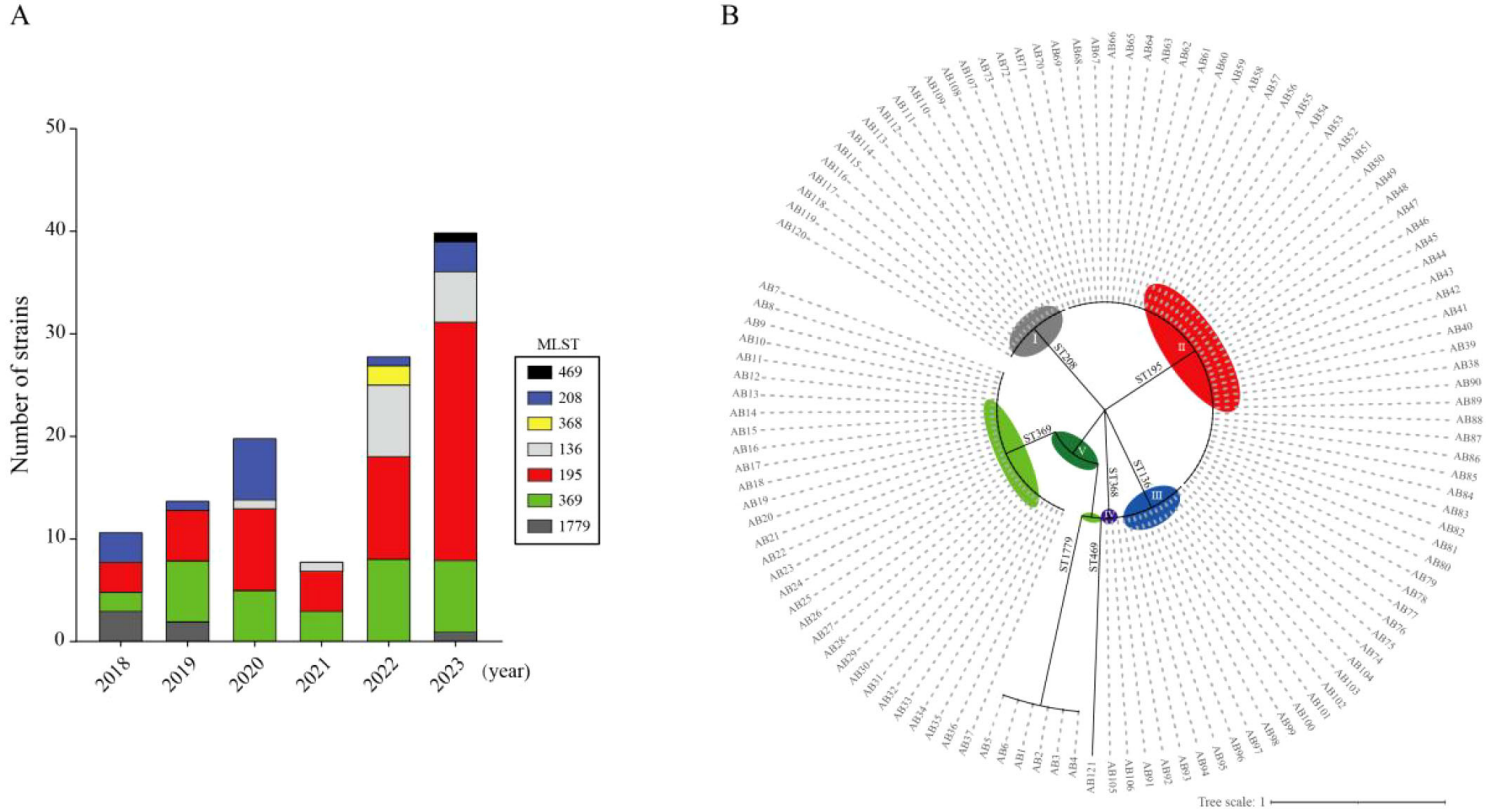
The analysis of MLST typing and the phylogenetic tree of CRAB from 2018 to 2023 is presented. **(A)** A total of seven ST types have been identified, and their annual distribution is illustrated. **(B)** The dendrogram of the phylogenetic tree constructed from 121 CRAB strains using GrapeTree software is presented. The colors gray, red, blue, purple, and dark green represent Clades I, II, III, IV, and V, respectively. Additionally, light green indicates two subgroups within Clade V.

MLST was determined by analyzing seven housekeeping genes of *A. baumannii* (*cpn60, fusA, gltA, pyrG, recA, rplB*, and *rpoB*) through PCR amplification and Sanger sequencing. The results indicated the presence of seven ST types: ST195 with 53 strains (43.8%), ST369 with 31 strains (25.6%), ST136 with 14 strains (11.5%), ST208 with 14 strains (11.5%), ST1779 with 6 strains (4.9%), ST368 with 2 strains (1.6%), and ST469 with 1 strain (0.8%). The annual distribution indicates that the highest number of detected CRAB cases occurred in 2022 (n=28) and 2023 (n=40), predominantly represented by ST195 and ST369 (**Figure 1A**). This suggests a rising trend in the prevalence of CRAB. The genetic backgrounds of all strains are closely related, as evidenced by phylogenetic tree analysis, which reveals five distinct clades: Clade I (ST208), Clade II (ST195), Clade III (ST136), Clade IV (ST368), and Clade V (ST1779, ST469, ST369) (**Figure 1B**).

### Clinical characteristic differences between ST195 positive patients and non-ST195 positive patients

MLST analysis revealed that ST195 is predominant in the region. Consequently, we further investigated the clinical characteristics of patients with ST195 in comparison to those with other sequence types (non-ST195). The results indicated no significant differences in age, gender, or length of hospital stay between the ST195 group and the non-ST195 group. However, the recovery rate for patients with ST195 was 30.2%, significantly lower than the 54.4% recovery rate observed in the non-ST195 group (P = 0.008). This finding suggests a poorer prognosis for patients infected with ST195. Regarding biochemical indicators, no significant differences were found between the two groups in terms of procalcitonin, hemoglobin, ESR, lymphocyte count, and neutrophil count levels. However, the ST195 group exhibited significantly higher levels of C-reactive protein (CRP) and interleukin-6 (IL-6), as well as elevated white blood cell (WBC) and platelet counts compared to the non-ST195 group, indicating a more severe immune response. Additionally, there were no significant differences in the distribution of underlying diseases, such as hypertension and diabetes, between the two groups. All results are summarized in **Table 3**.

**Table 3:** Differences in clinical characteristics of patients with ST195 and non-ST195 CRAB strains.

Clinical features	ST195 (*n* = 53)	non-ST195 (*n* = 68)	*p* (*z*/*χ*^2^)
Age, year, median (IQR)	59 (34.5-70.5)	63.5 (52.25-70.75)	0.133 (-1.5)
Gender, male, N (%)	43 (81.1%)	51 (75%)	0.422 (0.646)
Hospitalization stay, days, median (IQR)	27 (15.2-44)	21 (15-34.25)	0.272 (-1.098)
Outcome, recovered, N (%)	16 (30.2%)	37 (54.4%)	0.008* (7.1)
Laboratory features			
CRP, mg/L, median (IQR)	46.3 (20-82)	24.5 (9.01-57.07)	0.019* (-2.343)
WBC, 10^9/L, median (IQR)	13.95 (8.42-17.96)	10.03 (7.35-14.66)	0.011* (-2.544)
NEUT, %, median (IQR)	84.6 (79.4-89.15)	83.75 (78-89.93)	0.629 (-0.483)
LYM, %, median (IQR)	8.54 (5.68-14.32)	7.66 (4.61-12.9)	0.344 (-0.946)
ESR, mm/h, median (IQR)	25 (12-48)	26 (12.25-45.75)	0.849 (-0.191)
Hemoglobin, g/L, median (IQR)	108 (90-126.5)	112 (93.25-136.75)	0.261 (-1.123)
Platelet count, 10^9/L, median (IQR)	254 (145-305)	177 (121.5-269.75)	0.022* (-2.291)
PCT, ng/mL, median (IQR)	0.46 (0.17-3.4)	0.625 (0.22-2.27)	0.9 (-0.125)
IL-6, pg/mL, median (IQR)	165.86 (67.77-252.1)	76.5 (29.95-151.4)	0.001* (-3.367)
Combined underlying diseases, N (%)			
Hypertension	15 (28.3%)	13 (19.11%)	0.235 (1.41)
Diabetes	6 (11.3%)	9 (13.2%)	0.75 (0.1)

There is a statistically significant difference in the recovered rates, CRP, WBC, platelet count, and IL-6 levels between patients with ST195 and non-ST195 CRAB strains. Asterisks (*) denote statistically significant values. CRP, c-reactive protein; WBC, white blood cell; NEUT, neutrophil; LYM, lymphocyte; ESR, erythrocyte sedimentation rate; PCT, procalcitonin; IL-6, interleukin-6.

### Resistance analysis of CRAB strains

We conducted antimicrobial susceptibility testing on 121 strains of CRAB strainsagainst 15 antibiotics. The results indicated that all strains were resistant to seven antibiotics: imipenem (IPM), meropenem (MEM), piperacillin (PIP), ticarcillin-clavulanate (TCC), piperacillin-tazobactam (PIP-TAZ), ciprofloxacin (CIP), and ceftazidime (CAZ), thereby confirming that these 121 CRAB strains are multidrug-resistant. The resistance rates for cefepime (FEP), tobramycin (TOB), levofloxacin (LVX), and cefoperazone-sulbactam (CFP-SB) were recorded at 96.69%, 93.39%, 86.78%, and 83.47%, respectively. Resistance to trimethoprim-sulfamethoxazole (TMP-SMX) was observed at 48.76%. Additionally, the resistance rates for minocycline (MNO) and tigecycline (TGC) were found to be 28.93% and 20.66%, respectively. All strains were sensitive to colistin (COL). Although there were no differences in resistance to 13 antibiotics between ST195 and non-ST195 CRAB, the proportion of ST195 strains resistant to CFP-SB was significantly higher (100% vs 70.6%, p<0.001), while the proportions resistant to TMP-SMX and TGC were lower (28.3% vs 66.2%, p<0.001; 11.3% vs 27.9%, p=0.025). All results are detailed in **Table 4**.

**Table 4:** Drug resistance of clinical CRAB isolates.

Antibiotics	All CRAB (%)	ST195 (*n* = 53)	non-ST195 (*n* = 68)	*p* (*χ*^2^)
IPM	121 (100%)	53 (100)	68 (100)	–
MEM	121 (100%)	53 (100)	68 (100)	–
MNO	35 (28.93%)	14 (26.4)	21 (30.8)	–
PIP	121 (100%)	53 (100)	68 (100)	–
TCC	121 (100%)	53 (100)	68 (100)	–
TOB	113 (93.39%)	49 (92.4)	64 (94.1)	–
PIP-TAZ	121 (100%)	53 (100)	68 (100)	–
LVX	105 (86.78%)	45 (84.9)	60 (88.2)	–
TMP-SMX	59 (48.76%)	15 (28.3)	45 (66.2)	<0.001* (17.092)
TGC	25 (20.66%)	6 (11.3)	19 (27.9)	0.025* (5.019)
CIP	121 (100%)	53 (100)	68 (100)	–
CAZ	121 (100%)	53 (100)	68 (100)	–
COL	0 (0%)	0 (0%)	0 (0%)	–
CFP-SB	101 (83.47%)	53 (100)	48 (70.6)	<0.001* (18.675)
FEP	117 (96.69%)	53 (100)	64 (94.1)	–

IPM, imipenem; MEM, meropenem; MNO, minocycline; PIP, piperacillin; TC, ticarcillin-clavulnic acid; TOB, tobramycin; PIP-TAZ, piperacillin-tazobactam; LVX, levofloxacin; TMP-SMX, trimethoprim-sulfamethoxazole; TGC, tigecycline; CIP, ciprofloxacin; CAZ, ceftazidime; COL, colistin; CFP-SB, cefoperazone-sulbactam; FEP, cefepime. Asterisks (*) denote statistically significant values.

### Features of resistance and virulence genes

The previous results indicate that these strains belong to multi-drug resistant CRAB, with over 50% of infected patients exhibiting a poor prognosis, suggesting the presence of specific virulence factors. Consequently, we conducted polymerase chain reaction (PCR) assays to detect the resistance and virulence genes carried by these strains. The results revealed that all strains harbored *bla*_OXA-51_ and *bla*_OXA-23_, with 10% additionally carrying *bla*_VIM_, while other resistance genes such as *bla*_NDM_, *bla*_IMP_, *bla*_KPC_, *bla*_OXA-24_, and *bla*_OXA-58_ were not detected. Virulence factors *ompA*, *adeH*, *pgaA*, *abal*, *basJ*, and *plcD* were identified in all 121 CRAB strains, with 98.35% carrying *csuA* and 76.03% carrying *ptK*. The presence of these resistance genes and virulence factors may account for the resistance and virulence exhibited by CRAB. All related results are detailed in **Table 5**.

**Table 5:** Resistance or virulence genes present in the CRAB strains.

Genes	*n* (%)
*bla*_OXA-51_	121 (100%)
*bla*_OXA-23_	121 (100%)
*bla*_VIM_	10 (8.26%)
*ompA*	121 (100%)
*adeH*	121 (100%)
*pgaA*	121 (100%)
*abaI*	121 (100%)
*basJ*	121 (100%)
*plcD*	121 (100%)
*csuA*	119 (98.35)
*ptK*	92 (76.03%)
*bla*_OXA-24_	0 (0%)
*bla*_OXA-58_	0 (0%)
*bla*_NDM_	0 (0%)
*bla*_IMP_	0 (0%)
*bla*_KPC_	0 (0%)
*bla*_GES_	0 (0%)

### *Galleria mellonella* infection experiment

**Figure 2:**
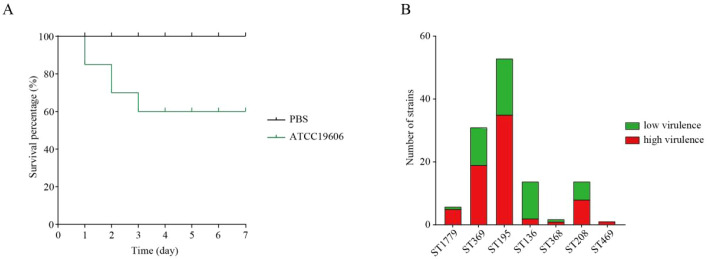
The *Galleria mellonella* infection assay was conducted to evaluate the pathogenicity of clinical carbapenem-resistant *Acinetobacter baumannii* (CRAB), with phosphate-buffered saline (PBS) serving as the negative control and ATCC19606 as the non-hypervirulent strain control. **(A)** The survival curves of *Galleria mellonella* injected with PBS and ATCC19606 over a period of 7 days are presented. **(B)** Statistical results regarding the prevalence of high-virulence and low-virulence strains among different sequence types (ST) of CRAB are summarized.

*Galleria mellonella* (*G. mellonella*) is frequently utilized to examine host–pathogen interactions due to its low cost, straight-forward handling, and an innate immune response comparable to that of mammals. Consequently, numerous studies have employed the *G. mellonella* infection model to investigate the pathogenic mechanisms of *A. baumannii* (Bjorge and Baillie, 1985; Tao et al., 2021; Sharma et al., 2025). In our study, we utilized the *G. mellonella* infection model to assess the CRAB strains carrying multiple virulence factors. The results show that the survival rate of *G. mellonella* larvae injected with PBS was 100% after 7 days, while the non-hypervirulent control ATCC19606 had a survival rate of 60% (**Figure 2A**). We define strains with a survival rate lower than 60%, corresponding to the survival rate of *G. mellonella* infected with ATCC19606, as hypervirulent CRAB. The results demonstrated that 71 strains (58.6%) fell into the high virulence category, comprising 35 strains of ST195, 19 strains of ST369, 8 strains of ST208, 5 strains of ST1779, 2 strains of ST136, 1 strain of ST368, and 1 strain of ST469. Notably, the proportions of high virulence strains were particularly high in ST1779, ST195, ST369, and ST208, at 83.3%, 66%, 61.3%, and 57.1%, respectively (**Figure 2B**). Clinical data indicated that 56.2% of patients experienced poor prognoses, potentially linked to the high virulence characteristics of these strains.

## Discussion

*Acinetobacter baumannii* (*A. baumannii*), a prevalent opportunistic pathogen responsible for hospital-acquired infections, is capable of causing a range of diseases, primarily lung and bloodstream infections (Dijkshoorn et al., 2007; Alsan and Klompas, 2010; McConnell et al., 2013). Multiple studies have demonstrated the presence of *A. baumannii* in BALF from patients suffering from lung infections (Jiang et al., 2024; Liu et al., 2024). Epidemiological data further indicate that *A. baumannii* is a significant pathogen associated with severe pulmonary infections, as evidenced by bacterial culture results from BALF samples collected from 4,080 children in Italy between January 2017 and December 2022 (Yu et al., 2025). Consequently, the detection of *A. baumannii* in BALF is crucial for the accurate diagnosis and effective treatment of lung infections. Furthermore, the resistance of this strain to carbapenem antibiotics has led to the emergence of carbapenem-resistant *A. baumannii* (CRAB), with its primary resistance mechanism closely linked to the production of OXA-type beta-lactamases (Poirel and Nordmann, 2006; Müller et al., 2023). Our study revealed that 121 strains of CRAB isolated from BALF at a hospital in Ningxia carried the *bla*_OXA-23_ and *bla*_OXA-51_ genes. Multilocus sequence typing (MLST) analysis indicated that sequence types ST195 and ST369 were the most prevalent, both of which belong to Global Clone 2 (GC2) that has contributed to the global dissemination of CRAB (Wang et al., 2023). The results of the Galleria mellonella infection assay revealed that 71strains (58.6%) exhibited high virulence phenotype. Additionally, virulence factors such as *ompA, adeH, pgaA, abal, basJ*, and *plcD* were detected in all tested strains, confirming an evolutionary trend towards high virulence in CRAB, which poses a serious threat to clinical treatment and patient prognosis.

The patients infected with CRAB included in this study were primarily sourced from the Intensive Care Unit (ICU) and had undergone invasive surgeries, resulting in a mortality rate of 56.2%. Lim et al. reported that the mortality rate for pneumonia caused by *A. baumannii* infections can reach as high as 42.6%, underscoring the significant challenge pneumonia from *A. baumannii* poses to patient survival (Mohd Sazlly Lim et al., 2019). Prior research has indicated that infections caused by CRAB are linked to elevated morbidity and mortality rates (Esterly et al., 2011; Quyen et al., 2025). Our phylogenetic analysis reveals that all strains share a closely related genetic background, suggesting the potential for clonal transmission among ICU patients over varying periods. Moreover, patients infected with ST195 exhibit a more severe immune response and poorer outcomes compared to those infected with non-ST195 strains, indicating a correlation between ST195 and unfavorable clinical prognosis. Research indicates that clinically isolated ST195 CRAB exhibits high virulence (Ali et al., 2017; Park et al., 2023), and patients infected with ST195 experience severe infections accompanied by organ damage (Niu et al., 2023). This suggests that the shift in dominant sequence types of CRAB strains from ST191 to ST195 in China may be attributed to ST195’s adaptation through the acquisition of higher virulence.

These CRAB strains exhibit not only resistance to carbapenem antibiotics but also a complete resistance (100%) to piperacillin, tigecycline, piperacillin-tazobactam, ciprofloxacin, and ceftazidime. Furthermore, they demonstrate high resistance rates (80%) to cefepime, tobramycin, levofloxacin, and cefoperazone-sulbactam, while showing low resistance to minocycline and tigecycline, and are fully sensitive (100%) to colistin. These findings are consistent with a study conducted in Guangdong Province, China (Karampatakis et al., 2024), where the majority of CRAB isolates, with the exception of tigecycline (49.2% resistant) and colistin (100% sensitive), demonstrated resistance to all tested antibiotics. This indicates that a significant proportion of *A. baumannii* infections among hospitalized respiratory patients are caused by multidrug-resistant strains, resulting in limited treatment options. Polymyxin and tigecycline remain the most effective antimicrobial agents against CRAB, multidrug-resistant *Acinetobacter baumannii* (MDR-AB), and extensively drug-resistant *A. baumannii* (XDR-AB) (Liu et al., 2025a). However, widespread resistance to tigecycline has been reported globally (Sun et al., 2013; Nowak et al., 2017; Qian et al., 2023), particularly due to their overuse in countries such as Germany (Eigenbrod et al., 2019), Egypt (Jalal et al., 2021), Greece (Papadopoulou et al., 2024). Aside from cefoperazone-sulbactam, trimethoprim-sulfamethoxazole, and tigecycline, no significant differences in antibiotic resistance have been observed between ST195 and non-ST195 CRAB strains in our study.

OXA-type hydrolases are the primary mechanism underlying CRAB, with all isolates from the Ningxia region harboring the *bla*_OXA-51_ and *bla*_OXA-23_ genes. Additionally, approximately 10% of these isolates carry the *bla*_VIM_ gene. Notably, other resistance genes such as *bla*_NDM_, *bla*_IMP_, *bla*_KPC_, *bla*_OXA-24_, and *bla*_OXA-58_ were not detected in this population. The *bla*_OXA-51_ gene is known to be a chromosomal intrinsic gene of *A. baumannii* (Turton et al., 2006), while *bla*_OXA-23_ is recognized as the most prevalent carbapenemase gene among CRAB isolates (Abbott et al., 2013; Fouad et al., 2013; Kuo et al., 2018; Huang et al., 2023). The presence of *bla*_OXA-51_ and *bla*_OXA-23_ genes is likely a significant factor contributing to the resistance of *A. baumannii* in hospital settings (Guo et al., 2022). Furthermore, the dissemination of carbapenemase-encoding genes is largely attributed to their association with integrons and mobile elements, such as *ISAba1*, *ISAba4*, and *ISAba125* (Mugnier et al., 2010; Lopes and Amyes, 2012; Nigro and Hall, 2015, 2016). However, the presence of these elements in the CRAB isolates in our study requires further verification.

In addition to multidrug resistance, CRAB also exhibits a high virulence, resulting in increased morbidity and mortality associated with the virulence factors they possess. These factors include the outer membrane gene *ompA*, which is involved in the invasion of bronchial epithelial cells (Choi et al., 2008), the efflux pump gene *adeH* (Choi et al., 2009), the biofilm formation related gene *pgaA* (Geisinger and Isberg, 2017), the quorum sensing system related gene *abaI* (Shan et al., 2022), the iron transporter synthesis gene *basJ* (Sheldon and Skaar, 2020), secretion system genes *plcD* and *csuA* involved in biofilm growth and fimbrial assembly (Zarrilli, 2016), and the membrane polysaccharide synthesis-related gene *ptk* (Ibrahim et al., 2021). PCR results indicated that a majority (76.3%) of resistant strains harbor these genes, demonstrating that these CRAB strains belong to hypervirulent CRAB. This was further evidenced by experiments utilizing *Galleria mellonella*, where 71 out of 121 strains (58.6%) were identified as hv-CRAB. The observed differences in mortality rates among strains carrying identical virulence factors in *Galleria mellonella* may be attributed to the mRNA expression levels of these factors, necessitating further validation. Seven highly virulent CRAB strains were isolated from hospitalized patients in central-southern China, and the mortality rate of patients infected with these strains averaged 42.9% within 7 days (Li et al., 2020). This indicates that CRAB isolates are increasingly multidrug-resistant and virulent, and the synergistic effect of these two phenotypes may contribute to treatment failure.

ST191 was the predominant CRAB isolate in China before 2018; however, ST195 emerged as the most prevalent ST type in China in 2018 (Park et al., 2023). Moreover, there are regional differences in the prevalence of STs across various parts of China. ST191 and ST195 are prevalent in Beijing, while ST208 and ST368 are found in Chongqing, and ST92 is present in Henan Province (Guo et al., 2016; Huang et al., 2017; Ning et al., 2017). In Ningxia, ST195 and ST369 dominate, with ST369 from China accounting for over 50% of the global epidemic ST369 clone (Huang et al., 2023). Additionally, other sequence types belonging to CC2, such as ST208, ST136, ST469, and ST368, have also been identified in our study area. We discovered another rare sequence type, ST1779, which has only been reported in Jilin and Beijing, with no occurrences in other locations according to the PubMLST database. CRAB isolates exhibiting a high virulent phenotype were observed across all STs identified in the study, suggesting that virulence genes may be widely disseminated and transferred among different strains, thereby necessitating further experimental validation.

However, this study has several limitations. First, the lack of follow-up data on the infected patients significantly restricts the clinical significance of our findings. Secondly, although we observed that most CRAB strains in this region exhibit multidrug resistance and high virulence phenotypes, there is a notable absence of genetic information analysis; we plan to incorporate whole-genome sequencing in future research. Thirdly, in addition to PCR detection of virulence genes and *Galleria mellonella* infection assays, further experiments are necessary to substantiate the virulence phenotypes, including serum resistance and mouse infection models.

## Conclusion

This study investigates the clinical characteristics, drug resistance, and virulence phenotypes of CRAB sourced from bronchoalveolar lavage fluid in Ningxia region from January 2018 to December 2023. The results indicate that the predominant sequence types are ST195 and ST369, with the rare ST1779 also identified. Patients infected with ST195 exhibited more severe immune responses and poorer prognoses compared to those infected with non-ST195 strains. In clinical treatment, it is essential to closely monitor the vital signs of patients infected with the ST195 CRAB strain and to make corresponding clinical decisions based on the trajectory of these changes. Most strains displayed high virulence phenotypes and harbored a significant number of resistance and virulence genes, suggesting that high-virulence CRAB has begun to spread in this region. This finding underscores the necessity for close monitoring and the development of appropriate preventive measures to avert further dissemination.

## Data Availability

The original contributions presented in the study are included in the article/supplementary material. Further inquiries can be directed to the corresponding author.

## References

[B1] Abbott I. Cerqueira G. M. Bhuiyan S. Peleg A. Y. (2013). Carbapenem resistance in Acinetobacter baumannii: laboratory challenges, mechanistic insights and therapeutic strategies. Expert Rev. Anti Infect. Ther. 11, 395–409. doi: 10.1586/eri.13.21, PMID: 23566149

[B2] Ali H. M. Salem M. Z. M. El-Shikh M. S. Megeed A. A. Alogaibi Y. A. Talea I. A. (2017). Investigation of the virulence factors and molecular characterization of the clonal relations of multidrug-resistant Acinetobacter baumannii isolates. J. AOAC Int. 100, 152–158. doi: 10.5740/jaoacint.16-0139, PMID: 27765082

[B3] Alsan M. Klompas M. (2010). Acinetobacter baumannii: an emerging and important pathogen. J. Clin. Outcomes Manag 17, 363–369. 26966345 PMC4782967

[B4] Bartual S. G. Seifert H. Hippler C. Luzon M. A. D. Wisplinghoff H. Rodríguez-Valera F. (2005). Development of a multilocus sequence typing scheme for characterization of clinical isolates of Acinetobacter baumannii. J. Clin. Microbiol. 43, 4382–4390. doi: 10.1128/JCM.43.9.4382-4390.2005, PMID: 16145081 PMC1234098

[B5] Bjorge S. M. Baillie T. A. (1985). Inhibition of medium-chain fatty acid beta-oxidation *in vitro* by valproic acid and its unsaturated metabolite, 2-n-propyl-4-pentenoic acid. Biochem. Biophys. Res. Commun. 132, 245–252. doi: 10.1016/0006-291x(85)91014-9, PMID: 3933498

[B6] Brown S. Amyes S. G. B. (2005). The sequences of seven class D beta-lactamases isolated from carbapenem-resistant Acinetobacter baumannii from four continents. Clin. Microbiol. Infect. 11, 326–329. doi: 10.1111/j.1469-0691.2005.01096.x, PMID: 15760431

[B7] Chang Y. Luan G. Xu Y. Wang Y. Shen M. Zhang C. . (2015). Characterization of carbapenem-resistant Acinetobacter baumannii isolates in a Chinese teaching hospital. Front. Microbiol. 6. doi: 10.3389/fmicb.2015.00910, PMID: 26388854 PMC4555021

[B8] Choi A. H. K. Slamti L. Avci F. Y. Pier G. B. Maira-Litrán T. (2009). The pgaABCD locus of Acinetobacter baumannii encodes the production of poly-beta-1-6-N-acetylglucosamine, which is critical for biofilm formation. J. Bacteriol 191, 5953–5963. doi: 10.1128/JB.00647-09, PMID: 19633088 PMC2747904

[B9] Choi C. H. Lee J. S. Lee Y. C. Park T. I. Lee J. C. (2008). Acinetobacter baumannii invades epithelial cells and outer membrane protein A mediates interactions with epithelial cells. BMC Microbiol. 8, 216. doi: 10.1186/1471-2180-8-216, PMID: 19068136 PMC2615016

[B10] Clinical and Laboratory Standards Institute (2020). Performance standards for antimicrobial susceptibility testing, (Wayne, PA, USA: CLSI) 30th ed, M100.

[B11] Dijkshoorn L. Nemec A. Seifert H. (2007). An increasing threat in hospitals: multidrug-resistant Acinetobacter baumannii. Nat. Rev. Microbiol. 5, 939–951. doi: 10.1038/nrmicro1789, PMID: 18007677

[B12] Doughty E. L. Liu H. Moran R. A. Hua X. Ba X. Guo F. . (2023). Endemicity and diversification of carbapenem-resistant Acinetobacter baumannii in an intensive care unit. Lancet Reg. Health West Pac 37, 100780. doi: 10.1016/j.lanwpc.2023.100780, PMID: 37693864 PMC10485671

[B13] Eigenbrod T. Reuter S. Gross A. Kocer K. Günther F. Zimmermann S. . (2019). Molecular characterization of carbapenem-resistant Acinetobacter baumannii using WGS revealed missed transmission events in Germany from 2012-15. J. Antimicrob. Chemother. 74, 3473–3480. doi: 10.1093/jac/dkz360, PMID: 31504593

[B14] Escribano Montaner A. García de Lomas J. Villa Asensi J. R. Asensio de la Cruz O. de la Serna Blázquez O. Santiago Burruchaga M. . (2018). Bacteria from bronchoalveolar lavage fluid from children with suspected chronic lower respiratory tract infection: results from a multi-center, cross-sectional study in Spain. Eur. J. Pediatr. 177, 181–192. doi: 10.1007/s00431-017-3044-3, PMID: 29285648 PMC5758651

[B15] Esterly J. S. Griffith M. Qi C. Malczynski M. Postelnick M. J. Scheetz M. H. (2011). Impact of carbapenem resistance and receipt of active antimicrobial therapy on clinical outcomes of Acinetobacter baumannii bloodstream infections. Antimicrob. Agents Chemother. 55, 4844–4849. doi: 10.1128/AAC.01728-10, PMID: 21825287 PMC3186964

[B16] Fouad M. Attia A. S. Tawakkol W. M. Hashem A. M. (2013). Emergence of carbapenem-resistant Acinetobacter baumannii harboring the OXA-23 carbapenemase in intensive care units of Egyptian hospitals. Int. J. Infect. Dis. 17, e1252–e1254. doi: 10.1016/j.ijid.2013.07.012, PMID: 24084245

[B17] Geisinger E. Isberg R. R. (2017). Interplay between antibiotic resistance and virulence during disease promoted by multidrug-resistant bacteria. J. Infect. Dis. 215, S9–S17. doi: 10.1093/infdis/jiw402, PMID: 28375515 PMC5853982

[B18] Gomaa F. A. M. Helal Z. H. Khan M. I. (2017). High Prevalence of blaNDM-1, blaVIM, qacE, and qacEΔ1 Genes and Their Association with Decreased Susceptibility to Antibiotics and Common Hospital Biocides in Clinical Isolates of Acinetobacter baumannii. Microorganisms 5, 18. doi: 10.3390/microorganisms5020018, PMID: 28417918 PMC5488089

[B19] Guo X. Cao Z. Zhang Z. (2016). Dissemination and evolution of blaOXA-23 gene among carbapenem-resistant Acinetobacter baumannii isolates from central China. J. Infect. Dev. Ctries 10, 445–448. doi: 10.3855/jidc.7498, PMID: 27131012

[B20] Guo H.-B. Huang H.-L. Li Y.-Y. (2022). Detection and homology analysis of carbapenem resistant Acinetobacter baumannii resistance gene. Front. Cell Infect. Microbiol. 12. doi: 10.3389/fcimb.2022.987260, PMID: 36683680 PMC9853024

[B21] He S. Li Z. Yang Q. Quan M. Zhao L. Hong Z. (2020). Resistance trends among 1,294 nosocomial Acinetobacter baumannii strains from a tertiary general hospital in China 2014 - 2017. Clin. Lab. 66. doi: 10.7754/Clin.Lab.2019.190629, PMID: 32162874

[B22] Huang Y. Ali M. R. Li W. Wang W. Dai Y. Lu H. . (2023). Epidemiological characteristics of multidrug-resistant Acinetobacter baumannii ST369 in Anhui, China. mSystems 8, e0073123. doi: 10.1128/msystems.00731-23, PMID: 37655924 PMC10654100

[B23] Huang G. Peng Y. Yang Y. Tang C. Fu Y. (2017). Multilocus sequence typing and molecular characterization of β-lactamase genes among Acinetobacter baumannii isolates in a burn center. Burns 43, 1473–1478. doi: 10.1016/j.burns.2017.03.020, PMID: 28461077

[B24] Hubloher J. J. van der Sande L. Schaudinn C. Müller V. Averhoff B. (2023). The Tol-Pal system of Acinetobacter baumannii is important for cell morphology, antibiotic resistance and virulence. Int. Microbiol. 26, 543–550. doi: 10.1007/s10123-022-00319-9, PMID: 36648597 PMC10397113

[B25] Ibrahim S. Al-Saryi N. Al-Kadmy I. M. S. Aziz S. N. (2021). Multidrug-resistant Acinetobacter baumannii as an emerging concern in hospitals. Mol. Biol. Rep. 48, 6987–6998. doi: 10.1007/s11033-021-06690-6, PMID: 34460060 PMC8403534

[B26] Jalal D. Elzayat M. G. Diab A. A. El-Shqanqery H. E. Samir O. Bakry U. . (2021). Deciphering multidrug-resistant Acinetobacter baumannii from a pediatric cancer hospital in Egypt. mSphere 6, e0072521. doi: 10.1128/mSphere.00725-21, PMID: 34787450 PMC8597740

[B27] Jeon J. H. Lee J. H. Lee J. J. Park K. S. Karim A. M. Lee C.-R. . (2015). Structural basis for carbapenem-hydrolyzing mechanisms of carbapenemases conferring antibiotic resistance. Int. J. Mol. Sci. 16, 9654–9692. doi: 10.3390/ijms16059654, PMID: 25938965 PMC4463611

[B28] Jiang L. Han L. Zhong Y. Zhang M. Li J. Rao G. . (2024). High utility of bronchoalveolar lavage fluid metagenomic next-generation sequencing approach for etiological diagnosis of pneumonia. BMC Infect. Dis. 24, 1232. doi: 10.1186/s12879-024-10108-6, PMID: 39488700 PMC11531162

[B29] Jiang L. Liang Y. Yao W. Ai J. Wang X. Zhao Z. (2019). Molecular epidemiology and genetic characterisation of carbapenem-resistant Acinetobacter baumannii isolates from Guangdong Province, South China. J. Glob Antimicrob. Resist. 17, 84–89. doi: 10.1016/j.jgar.2018.11.002, PMID: 30445207

[B30] Karampatakis T. Tsergouli K. Behzadi P. (2024). Pan-genome plasticity and virulence factors: A natural treasure trove for Acinetobacter baumannii. Antibiotics (Basel) 13, 257. doi: 10.3390/antibiotics13030257, PMID: 38534692 PMC10967457

[B31] Kuo S.-C. Huang W.-C. Huang T.-W. Wang H.-Y. Lai J.-F. Chen T.-L. . (2018). Molecular Epidemiology of Emerging blaOXA-23-Like- and blaOXA-24-Like-Carrying Acinetobacter baumannii in Taiwan. Antimicrob. Agents Chemother. 62, e01215–e01217. doi: 10.1128/AAC.01215-17, PMID: 29311067 PMC5826164

[B32] Lee C.-R. Lee J. H. Park M. Park K. S. Bae I. K. Kim Y. B. . (2017). Biology of Acinetobacter baumannii: pathogenesis, antibiotic resistance mechanisms, and prospective treatment options. Front. Cell Infect. Microbiol. 7. doi: 10.3389/fcimb.2017.00055, PMID: 28348979 PMC5346588

[B33] Li J. Li Y. Cao X. Zheng J. Zhang Y. Xie H. . (2023). Genome-wide identification and oxacillinase OXA distribution characteristics of Acinetobacter spp. based on a global database. Front. Microbiol. 14. doi: 10.3389/fmicb.2023.1174200, PMID: 37323896 PMC10267304

[B34] Li J. Yu T. Luo Y. Peng J.-Y. Li Y.-J. Tao X.-Y. . (2020). Characterization of carbapenem-resistant hypervirulent Acinetobacter baumannii strains isolated from hospitalized patients in the mid-south region of China. BMC Microbiol. 20, 281. doi: 10.1186/s12866-020-01957-7, PMID: 32928115 PMC7489012

[B35] Lin M.-F. Lan C.-Y. (2014). Antimicrobial resistance in Acinetobacter baumannii: From bench to bedside. World J. Clin. cases 2, 787–814. doi: 10.12998/wjcc.v2.i12.787, PMID: 25516853 PMC4266826

[B36] Liu C. Chen K. Wu Y. Huang L. Fang Y. Lu J. . (2022). Epidemiological and genetic characteristics of clinical carbapenem-resistant Acinetobacter baumannii strains collected countrywide from hospital intensive care units (ICUs) in China. Emerg. Microbes Infect. 11, 1730–1741. doi: 10.1080/22221751.2022.2093134, PMID: 35730377 PMC9258068

[B37] Liu L. Liu B. Li L. Li Y. Zhou X. Li Q. (2025a). Impact of Antimicrobial Stewardship and Infection Prevention and Control Programmes on Antibiotic Usage and A. baumannii resistance: A 2016–2023 Multicentre Prospective Study. Infect. Drug Resist. 18, 679–692. doi: 10.2147/IDR.S505133, PMID: 39926172 PMC11806701

[B38] Liu Y. Wu W. Xiao Y. Zou H. Hao S. Jiang Y. (2024). Application of metagenomic next-generation sequencing and targeted metagenomic next-generation sequencing in diagnosing pulmonary infections in immunocompetent and immunocompromised patients. Front. Cell Infect. Microbiol. 14. doi: 10.3389/fcimb.2024.1439472, PMID: 39165919 PMC11333343

[B39] Liu Z. Zhang L. Zou J. (2025b). Epidemiological characteristics and antimicrobial resistance of pathogens isolated from blood cultures in southern Jiangxi, China 2020-2024. Front. Cell Infect. Microbiol. 15. doi: 10.3389/fcimb.2025.1727877, PMID: 41607523 PMC12835207

[B40] Lopes B. S. Amyes S. G. B. (2012). Role of ISAba1 and ISAba125 in governing the expression of blaADC in clinically relevant Acinetobacter baumannii strains resistant to cephalosporins. J. Med. Microbiol. 61, 1103–1108. doi: 10.1099/jmm.0.044156-0, PMID: 22499776

[B41] Lucidi M. Visaggio D. Migliaccio A. Capecchi G. Visca P. Imperi F. . (2024). Pathogenicity and virulence of Acinetobacter baumannii: Factors contributing to the fitness in healthcare settings and the infected host. Virulence 15, 2289769. doi: 10.1080/21505594.2023.2289769, PMID: 38054753 PMC10732645

[B42] McConnell M. J. Actis L. Pachón J. (2013). Acinetobacter baumannii: human infections, factors contributing to pathogenesis and animal models. FEMS Microbiol. Rev. 37, 130–155. doi: 10.1111/j.1574-6976.2012.00344.x, PMID: 22568581

[B43] Mea H. J. Yong P. V. C. Wong E. H. (2021). An overview of Acinetobacter baumannii pathogenesis: Motility, adherence and biofilm formation. Microbiol. Res. 247, 126722. doi: 10.1016/j.micres.2021.126722, PMID: 33618061

[B44] Mohd Sazlly Lim S. Zainal Abidin A. Liew S. M. Roberts J. A. Sime F. B. (2019). The global prevalence of multidrug-resistance among Acinetobacter baumannii causing hospital-acquired and ventilator-associated pneumonia and its associated mortality: A systematic review and meta-analysis. J. Infect. 79, 593–600. doi: 10.1016/j.jinf.2019.09.012, PMID: 31580871

[B45] Mugnier P. D. Poirel L. Naas T. Nordmann P. (2010). Worldwide dissemination of the blaOXA-23 carbapenemase gene of Acinetobacter baumannii. Emerg. Infect. Dis. 16, 35–40. doi: 10.3201/eid1601.090852, PMID: 20031040 PMC2874364

[B46] Müller C. Reuter S. Wille J. Xanthopoulou K. Stefanik D. Grundmann H. . (2023). A global view on carbapenem-resistant Acinetobacter baumannii. mBio 14, e0226023. doi: 10.1128/mbio.02260-23, PMID: 37882512 PMC10746149

[B47] Neves F. C. Clemente W. T. Lincopan N. Paião I. D. Neves P. R. Romanelli R. M. . (2016). Clinical and microbiological characteristics of OXA-23- and OXA-143-producing Acinetobacter baumannii in ICU patients at a teaching hospital, Brazil. Braz. J. Infect. Dis. 20, 556–563. doi: 10.1016/j.bjid.2016.08.004, PMID: 27620658 PMC9427641

[B48] Nigro S. Hall R. M. (2015). Distribution of the blaOXA-23-containing transposons Tn2006 and Tn2008 in Australian carbapenem-resistant Acinetobacter baumannii isolates. J. Antimicrob. Chemother. 70, 2409–2411. doi: 10.1093/jac/dkv102, PMID: 25881617

[B49] Nigro S. J. Hall R. M. (2016). Structure and context of Acinetobacter transposons carrying the oxa23 carbapenemase gene. J. Antimicrob. Chemother. 71, 1135–1147. doi: 10.1093/jac/dkv440, PMID: 26755496

[B50] Ning N.-Z. Liu X. Bao C.-M. Chen S.-M. Cui E.-B. Zhang J.-L. . (2017). Molecular epidemiology of bla OXA-23 -producing carbapenem-resistant Acinetobacter baumannii in a single institution over a 65-month period in north China. BMC Infect. Dis. 17, 14. doi: 10.1186/s12879-016-2110-1, PMID: 28056839 PMC5217423

[B51] Niu T. Guo L. Kong X. He F. Ru C. Xiao Y. (2023). Prevalent dominant Acinetobacter baumannii ST191/195/208 strains in bloodstream infections have high drug resistance and mortality. Infection Drug Resistance 16, 2417. doi: 10.2147/IDR.S403604, PMID: 37138832 PMC10149779

[B52] Nowak P. Paluchowska P. (2016). Acinetobacter baumannii: biology and drug resistance - role of carbapenemases. Folia Histochem Cytobiol 54, 61–74. doi: 10.5603/FHC.a2016.0009, PMID: 27270503

[B53] Nowak J. Zander E. Stefanik D. Higgins P. G. Roca I. Vila J. . (2017). High incidence of pandrug-resistant Acinetobacter baumannii isolates collected from patients with ventilator-associated pneumonia in Greece, Italy and Spain as part of the MagicBullet clinical trial. J. Antimicrob. Chemother. 72, 3277–3282. doi: 10.1093/jac/dkx322, PMID: 28961773 PMC5890771

[B54] Papadopoulou M. Deliolanis I. Polemis M. Vatopoulos A. Psichogiou M. Giakkoupi P. (2024). Characteristics of the genetic spread of Carbapenem-resistant Acinetobacter baumannii in a tertiary Greek hospital. Genes (Basel) 15, 458. doi: 10.3390/genes15040458, PMID: 38674392 PMC11050095

[B55] Park S. M. Suh J. W. Ju Y. K. Kim J. Y. Kim S. B. Sohn J. W. . (2023). Molecular and virulence characteristics of carbapenem-resistant Acinetobacter baumannii isolates: a prospective cohort study. Sci. Rep. 13, 19536. doi: 10.1038/s41598-023-46985-1, PMID: 37945745 PMC10636183

[B56] Poirel L. Nordmann P. (2006). Carbapenem resistance in Acinetobacter baumannii: mechanisms and epidemiology. Clin. Microbiol. Infect. 12, 826–836. doi: 10.1111/j.1469-0691.2006.01456.x, PMID: 16882287

[B57] Qian C. Ma Z. Feng L. Guo W. Han Y. Zhang Y. . (2023). Emergence of tet(X2) in Acinetobacter pittii confers clinical resistance to tigecycline. J. Antimicrob. Chemother. 78, 1543–1546. doi: 10.1093/jac/dkad133, PMID: 37141282

[B58] Quyen T. L. T. Hsieh Y.-C. Li S.-W. Wu L.-T. Liu Y.-Z. Pan Y.-J. (2025). Molecular epidemiology of carbapenem-resistant Acinetobacter baumannii group in Taiwan. mSphere 10, e0079324. doi: 10.1128/msphere.00793-24, PMID: 39745372 PMC11774041

[B59] Ramirez M. S. Bonomo R. A. Tolmasky M. E. (2020). Carbapenemases: Transforming Acinetobacter baumannii into a Yet More Dangerous Menace. Biomolecules 10, 720. doi: 10.3390/biom10050720, PMID: 32384624 PMC7277208

[B60] Ruan Z. Chen Y. Jiang Y. Zhou H. Zhou Z. Fu Y. . (2013). Wide distribution of CC92 carbapenem-resistant and OXA-23-producing Acinetobacter baumannii in multiple provinces of China. Int. J. Antimicrob. Agents 42, 322–328. doi: 10.1016/j.ijantimicag.2013.06.019, PMID: 23988720

[B61] Shan W. Kan J. Cai X. Yin M. (2022). Insights into mucoid Acinetobacter baumannii: A review of microbiological characteristics, virulence, and pathogenic mechanisms in a threatening nosocomial pathogen. Microbiol. Res. 261, 127057. doi: 10.1016/j.micres.2022.127057, PMID: 35569319

[B62] Sharma S. Singh K. Chaurasiya A. Banerjee T. Singh R. Yadav G. . (2025). Comparative study of phenotypic and genotypic expression of virulence factors in colonizing and pathogenic carbapenem resistant Acinetobacter baumannii (CRAB). BMC Microbiol. 25, 13. doi: 10.1186/s12866-024-03727-1, PMID: 39799303 PMC11724464

[B63] Sheldon J. R. Skaar E. P. (2020). Acinetobacter baumannii can use multiple siderophores for iron acquisition, but only acinetobactin is required for virulence. PLoS Pathog. 16, e1008995. doi: 10.1371/journal.ppat.1008995, PMID: 33075115 PMC7595644

[B64] Shields R. K. Paterson D. L. Tamma P. D. (2023). Navigating available treatment options for Carbapenem-resistant Acinetobacter baumannii-calcoaceticus complex infections. Clin. Infect. Dis. 76, S179–S193. doi: 10.1093/cid/ciad094, PMID: 37125467 PMC10150276

[B65] Sun Y. Cai Y. Liu X. Bai N. Liang B. Wang R. (2013). The emergence of clinical resistance to tigecycline. Int. J. Antimicrob. Agents 41, 110–116. doi: 10.1016/j.ijantimicag.2012.09.005, PMID: 23127485

[B66] Tang X. Xiao M. Zhuo C. Xu Y. Zhong N. (2018a). Multi-level analysis of bacteria isolated from inpatients in respiratory departments in China. J. Thorac. Dis. 10, 2666–2675. doi: 10.21037/jtd.2018.04.46, PMID: 29997928 PMC6006077

[B67] Tang X. Zhuo C. Xu Y. C. Zhong N. S. (2018b). The composition and antimicrobial resistance of isolates from lower respiratory tract and blood in hospitalized patients in respiratory ward: a multicenter national study in China. Zhonghua Jie He He Hu Xi Za Zhi 41, 281–287. doi: 10.3760/cma.j.issn.1001-0939.2018.04.007, PMID: 29690684

[B68] Tao Y. Duma L. Rossez Y. (2021). Galleria mellonella as a Good Model to Study Acinetobacter baumannii Pathogenesis. Pathogens 10, 1483. doi: 10.3390/pathogens10111483, PMID: 34832638 PMC8623143

[B69] Tian C. Di L. Dong S. Tian X. Huang D. Zhao Y. . (2024). Whole genome sequencing and genomic characteristics analysis of carbapenem-resistant Acinetobacter baumannii clinical isolates in two hospitals in China. Infection Genet. Evol. 123, 105642. doi: 10.1016/j.meegid.2024.105642, PMID: 39013496

[B70] Turton J. F. Ward M. E. Woodford N. Kaufmann M. E. Pike R. Livermore D. M. . (2006). The role of ISAba1 in expression of OXA carbapenemase genes in Acinetobacter baumannii. FEMS Microbiol. Lett. 258, 72–77. doi: 10.1111/j.1574-6968.2006.00195.x, PMID: 16630258

[B71] Wang M. Ge L. Chen L. Komarow L. Hanson B. Reyes J. . (2023). Clinical outcomes and bacterial characteristics of Carbapenem-resistant Acinetobacter baumannii among patients from different global regions. Clin. Infect. Dis. 78, 248–258. doi: 10.1093/cid/ciad556, PMID: 37738153 PMC10874260

[B72] Wong D. Nielsen T. B. Bonomo R. A. Pantapalangkoor P. Luna B. Spellberg B. (2017). Clinical and pathophysiological overview of Acinetobacter infections: a century of challenges. Clin. Microbiol. Rev. 30, 409–447. doi: 10.1128/CMR.00058-16, PMID: 27974412 PMC5217799

[B73] Woodford N. Ellington M. J. Coelho J. M. Turton J. F. Ward M. E. Brown S. . (2006). Multiplex PCR for genes encoding prevalent OXA carbapenemases in Acinetobacter spp. Int. J. Antimicrob. Agents 27, 351–353. doi: 10.1016/j.ijantimicag.2006.01.004, PMID: 16564159

[B74] Yao L. Liu N. Guo Y. Zhuo C. Yang X. Wang Y. . (2024). Comparison of hypervirulent and non-hypervirulent Carbapenem-resistant Acinetobacter baumannii isolated from bloodstream infections: mortality, potential virulence factors, and combination therapy *in vitro*. Antibiotics (Basel) 13, 807. doi: 10.3390/antibiotics13090807, PMID: 39334982 PMC11428969

[B75] Yu M. Li M. Sun H. (2025). Dynamic analysis of the epidemiology and pathogen distribution of bronchoalveolar lavage fluid in children with severe pulmonary infection: a retrospective study. Ital J. Pediatr. 51, 18. doi: 10.1186/s13052-025-01859-2, PMID: 39875941 PMC11776209

[B76] Zarrilli R. (2016). Acinetobacter baumannii virulence determinants involved in biofilm growth and adherence to host epithelial cells. Virulence 7, 367–368. doi: 10.1080/21505594.2016.1150405, PMID: 26856342 PMC4871643

[B77] Zhang Z.-G. Chen F. Ou Y. (2017). Impact of an antimicrobial stewardship programme on antibiotic usage and resistance in a tertiary hospital in China. J. Clin. Pharm. Ther. 42, 579–584. doi: 10.1111/jcpt.12544, PMID: 28485087

[B78] Zhang P. Hao J. Zhang Y. Su J. Sun G. Xie J. . (2025). Understanding the clinical and molecular epidemiological characteristics of carbapenem-resistant Acinetobacter baumannii infections within intensive care units of three teaching hospitals. Ann. Clin. Microbiol. Antimicrob. 24, 2. doi: 10.1186/s12941-024-00766-4, PMID: 39806310 PMC11731405

